# Keystone Veterinary Medical Association

**Published:** 1896-03

**Authors:** W. L. Rhoads

**Affiliations:** Secretary


Secretary.

### KEYSTONE VETERINARY MEDICAL ASSOCIATION.

THE regular meeting was called to order by President J. R. Hart, Tuesday evening, February 11, at 8.30. The members present were, Drs. H. P. Eves, W. H. Hoskins, John R. Hart, Charles Lintz, H. J. McClellan, J. T. McAnulty, and W. L. Rhoads. The visitors were Drs. Thos. B. Rayner, F. S. Allen, J. O. George, J. McBirney, B. M. Underhill, and several students from the University Veterinary Department.

Dr. Hoskins spoke of the Army Bill, indorsed at the last meeting of the Association, and said though the committee appointed had done very little as a committee, they had, individually, been hard and earnestly working, going so far as to find just what interest was being manifested by the veterinarians in other States. He said, though the outlook was very favorable, he thought no veterinarian could consistently remain quiet and unheard-from while this issue was in the balance, as this was undoubtedly the time to secure army legislation, and the Senate was the battle-ground, as the House was comparatively safe.

It was announced that the State Board of Veterinary Medical Examiners would meet in Philadelphia, for the purpose of examining applicants, the third Monday and Tuesday of April.

Dr. Eves gave a very interesting talk on the contagious and infectious diseases of Delaware. He also told what noble work the Department of Agriculture was doing down there. He also brought out many valuable points in the art of securing legislation, principal among which was the maintaining of farmers' institutes. They thus educate the people and do away with political legislation, which must have emoluments or pe rquisites as a motive-power. This is becoming essential for the masses. While trying to convince themselves that the veterinarian must be looking for a snap, they lose sight of the fact that in the last ten years they, in Delaware, have lost five to six hundred thousand dollars' worth of stock. This amount would have furnished a complete sanitary police and have restabled all the cattle in the State under the most favorable circumstancess. Among those diseases he mentioned tuberculosis, anthrax, hydrophobia, ringworm in cattle, tetanus, meningitis, hog cholera, œstrus bovis, contagious abortion. Of tuberculosis, he said, in 1100 cows tested about 200 were condemned, and the post-mortems held on 90 per cent. of them proved the diagnosis to be correct. The tuberculin used was manufactured by Prof. Chester, of the Delaware Station, and it was found equally good as Koch's. They take temperature for two days before injecting and one day after injecting, and have always found the disease where there was a reaction. The Delaware Act does not make the test compulsory, but leaves it optional with the owner.

Those cattle showing rise of 8-10 per cent., or less, are not condemned, but put aside; those showing rise of 1 per cent. to 1½ per cent. are condemned. They believe the best way of eradicating it is by the Department making free tests and allowing only perfect animals to be bred. They have from 50 to 60 fatal cases of anthrax annually. This is found principally on the salt meadows, and is supposed to have been brought from Jersey, as tests prove the floodtide will bring débris from Jersey's infected district to this locality. They have inoculated about 850 cows, thus protecting them; have returned them to the infected pastures with a loss of but 2 head, six and eight weeks, respectively, after return, but have no cases this year, chargeable to vaccine. They use in each case 0.3 c.c. of virus of three grades: No. 1 kills white mice invariably and nothing else; No. 2 kills white mice and guinea-pigs in 60 per cent. of cases; No. 3 kills guinea-pigs invariably, but has never yet killed rabbits. Nos. 1 and 2 cause no disturbance whatever when injected into cows and calves; No. 3, in 12 to 15 instances in about 850 cases, caused marked œdema and loss of milk for twenty-four hours. They have had reports of death of 20 sheep near the affected district. The only law they have relating to anthrax is one forced through appropriating $500 for burial purposes. They have tested 14 cases of glanders at Middletown with mallein without reaction. He reported mare having discharge from nostrils for three years, suddenly became worse; tested with mallein and gave reaction of $103\frac{8}{10}°$. Her mate died of chronic peritonitis; showed cicatrices in the liver. The post-mortem in each case confirmed the diagnosis of glanders. Have had some mycotic enteritis which was supposed to be caused by feeding decaying turnips, as the trouble ceased when the feeding of the turnips was stopped, the other food being good. Yet other cattle (at the Station) fed on these turnips became quite fat.

The ringworm in cattle has been traced to the importation of York State calves, and if taken in time and treated with outward applications of tinct. iodi. gave little trouble. Tetanus is on the decrease; not nearly so prevalent as two years ago. The tetanin used is that of the Pasteur Institute. He also spoke of hog cholera, œstrus bovis, contagious abortion, which they treat successfully with hypodermic injections of carbolic acid.

Meningitis, within the last eight to ten years, has caused a loss of five to six thousand horses. They think this to be a chemical trouble caused by atmospheric changes, and suppose the death of the fungus causes ptomaines. Yet they have fed the supposed infectious food to young horses and have raised cultures of all fungous growths found on infected premises; have fed the pure cultures and have not been able to produce the disease.

They have no protective legislation covering hydrophobia. Have had 50 to 60 cases in cows traceable to dogs; 50 per cent. of these cases died.

Dr. McBirney exhibited two very interesting specimens, one being a section of pancreas from an ox. It consisted of a large duct filled with little white granules, most closely resembling homœopathic pills. This must contain 3 to 4 ounces of these granules. The other was a honeycomb piece of carbonate of lime, 2 by 1½ inches, taken from the brisket of an ox. Each of these animals were in good condition.

Drs. McAnulty and McClellan each reported cases of recovery in mules from cerebro-spinal meningitis. In the general discussion which followed these reports, Dr. Eves said he often found in this disease a great diversity of symptoms, many resembling influenza, some having putrid sore-throat; those having lost the power of deglutition, as a rule, have the highest temperature and the greatest congestion of the peritoneum. Dr. Hoskins thought the general sanitary surroundings affected the temperature and general condition. The most perfect surroundings being conducive to rise of temperature in early stages, while in poorer surroundings with a subnormal temperature, the acute symptoms vary according to parts from which poison is being absorbed. Those cases which are colicky in the outset seldom lose the power of deglutition. Dr. McAnulty cited case of azoturia to which he had given aloes, then placed on tr. juniper, buchu, and potash for two weeks, had then used strychnine, quinine, and iron; the patient did well till exercised, when the incoördination of the hind parts returned. Patient continually constipated, pulse weak, temperature 101°. He is fat and will not sweat. Dr. Hoskins thinks condition similar to locomotor ataxia of man. Dr. Allen advocates the use of sulph. strych., given until development of tetanic symptoms; best given after feeding.

Dr. B. M. Underhill reported case of rabies[1] in sorrel gelding nine years old, used as family horse. First showed symptoms two days before; would not trot, but persisted in galloping; following day snapped at boy; became vicious and would snap and kick when any one passed before him. Before being destroyed he became very irritable, kept up a constant champing of the jaws, causing a frothy saliva to exude. He would at all times obey commands of the attendants. After his destruction the brain and portions of the spinal cord were removed and taken to the University for experimental purposes. No history of his having been bitten, but dog formerly in his stable supposed to have gone mad.

Dr. Eves said he had treated, with the Pasteur virus, a horse that had been bitten by a rabid dog severely enough to draw blood several times, and had good results. He says cows seldom become violent, though they may be excitable. Dr. McBirney spoke of two-year-old colt having rabies from bite of wolf. After a vote of thanks to those who had so ably entertained them the Society adjourned to meet March 10, 1896. W. L. Rhoads,
Secretary.

